# Privacy-preserving aggregation of personal health data streams

**DOI:** 10.1371/journal.pone.0207639

**Published:** 2018-11-29

**Authors:** Jong Wook Kim, Beakcheol Jang, Hoon Yoo

**Affiliations:** 1 Department of Computer Science, Sangmyung University, Seoul, Korea; 2 Department of Electronic Engineering, Sangmyung University, Seoul, Korea; King Saud University, SAUDI ARABIA

## Abstract

Recently, as the paradigm of medical services has shifted from treatment to prevention, there is a growing interest in smart healthcare that can provide users with healthcare services anywhere, at any time, using information and communications technologies. With the development of the smart healthcare industry, there is a growing need for collecting large-scale personal health data to exploit the knowledge obtained through analyzing them for improving the smart healthcare services. Although such a considerable amount of health data can be a valuable asset to the smart healthcare fields, they may cause serious privacy problems if sensitive information of an individual user is leaked to outside users. Therefore, most individuals are reluctant to provide their health data to smart healthcare service providers for data analysis and utilization purpose, which is the biggest challenge in smart healthcare fields. Thus, in this paper, we develop a novel mechanism for privacy-preserving collection of personal health data streams that is characterized as temporal data collected at fixed intervals by leveraging local differential privacy (LDP). In particular, with the proposed approach, a data contributor uses a given privacy budget of LDP to report a small amount of salient data, which are extracted from an entire health data stream, to a data collector. Then, a data collector can effectively reconstruct a health data stream based on the noisy salient data received from a data contributor. Experimental results demonstrate that the proposed approach provides significant accuracy gains over straightforward solutions to this problem.

## Introduction

In recent years, with the development of information and communications technologies, smart healthcare services, focused on disease prevention and health promotion by continuously monitoring users’ health and providing real-time customized service, are receiving significant attention. The basic structure of smart healthcare is that service providers collect data generated by individual users in their daily lives and by medical institutions about patients and then provide customized advice and treatment to users based on the knowledge obtained through analyzing a large amount of collected data. With a rapidly aging society, increased medical burden due to chronic illnesses and increased interest in health due to abnormal climate conditions around the world, the demand for smart healthcare service is expected to continue to increase in the future.

Along with the development of the internet of things (IoT) technology, wearable devices based on IoT are being actively developed and used. In particular, the technology development of wearable devices that can continuously monitor human activity and bio-signals using sensors has played a major role in the development of the smart healthcare industry. For example, with the wide spread use of wearable devices having various bio sensors, it is possible to easily measure and monitor diverse health data such as blood glucose levels, blood pressure, oxygen saturation, heart rate, and body temperature of individuals. This, in turn, makes it possible to provide an alarm service, notifying in advance the risk of disease outbreak to users by collecting and analyzing vast amount of health data based on individual activities of daily living.

The development of the smart healthcare industry brings forth a need for collecting large-scale personal health data in order to leverage the knowledge obtained through analyzing such data for improving smart healthcare services. For example, Apple Health [1], Google Fit [2], and Samsung S-Health [3] aggregate vast amounts of health-related data using smartphones and wearable devices such as a smartwatch and smartband. A telecare medicine information system, which is widely used to provide remote medical care to a patient [4], continuously monitors and collects the patient’s health data through various physiological signal monitoring systems.

Serious concerns of data privacy have been raised in many areas over the past few years. One of the most representative areas is that of privacy in a cloud environment. In this environment, user data are typically stored on cloud servers, which are often outside of a trusted domain [5]. Even though a large collection of health data is a valuable asset to the smart healthcare field, similar data privacy concerns are raised. That is, indiscriminate collection of personal health data can cause significant privacy issues; sensitive information of individual users can be deduced by tracking and analyzing health data. Hence, most users do not agree to their health data being collected for the purposes of data analysis and utilization. This presents a major obstacle for the development of smart healthcare services.

**Fig 1:**
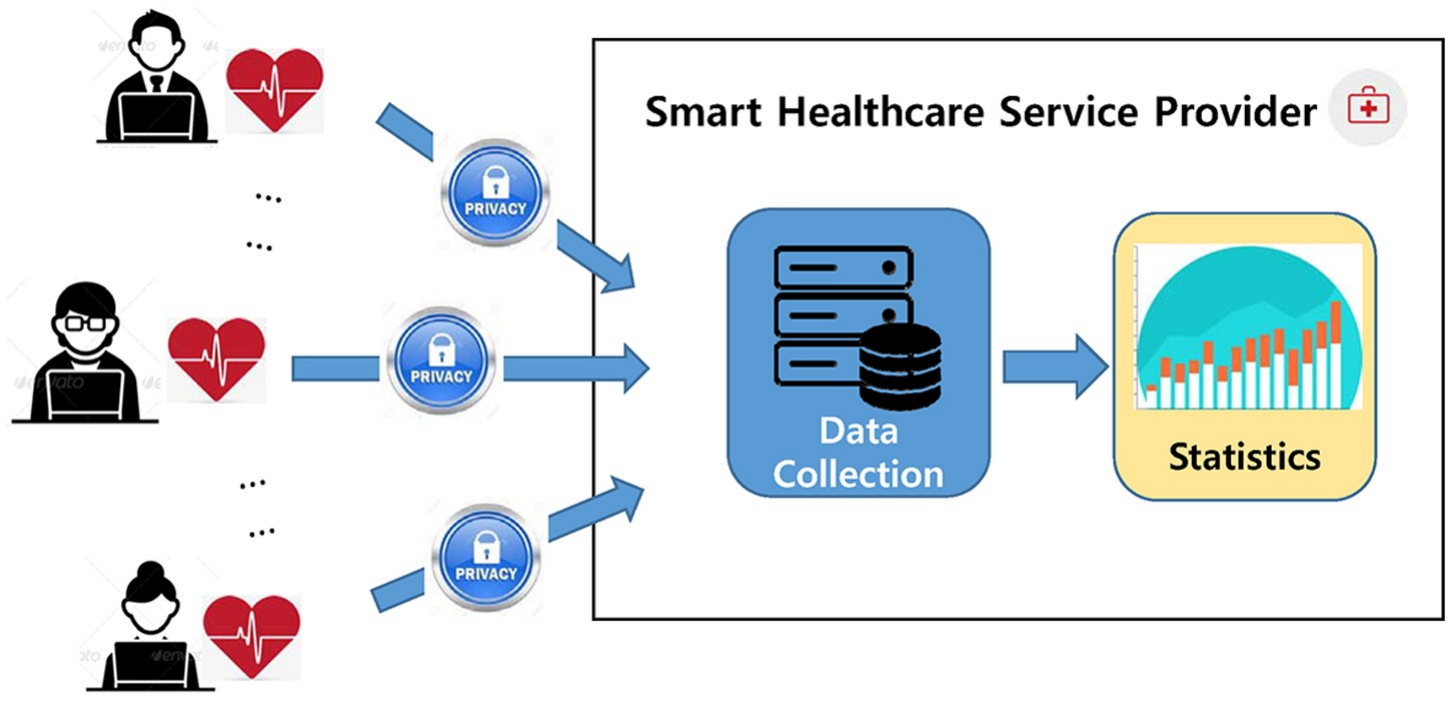
A motivational example.

Fig 1 illustrates the motivational scenario of this research where a smart healthcare service provider wants to collect and analyze a large volume of health data to obtain heart rate changes of individuals with desk jobs with the aim of enhancing the quality of healthcare service customized for them. However, considering that individual are reluctant to provide their sensitive health data, to support such a service provider’s requirement, it is essential to develop methods capable of collecting individuals’ health data, while preserving their privacy.

The goal in this paper is to develop a novel mechanism for privacy-preserving collection of individual health data streams generated from smart healthcare sensors by leveraging local differential privacy (LDP). LDP is a state-of-the-art approach that is used to protect individual privacy during the process of data collection [6]. The basic idea of the LDP is that a data contributor adds carefully designed random noises to the original data and sends the noisy data to a data collector, guaranteeing that the data contributor’s original data is not leaked during the data collection process. With the growing popularity of LDP, there have been extensive studies to leverage LDP for collecting individuals’ sensitive data, generated in diverse application domains, in a privacy-preserving manner [7–16]. However, these existing approaches focus on the collection of individual data represented as bit-strings where each bit corresponds to either 0 or 1, and thus they are not applicable for collecting individual health data that is usually represented as a stream (or time series). Thus, in this paper, we propose a novel mechanism for collecting individual health data, which is characterized as temporal data collected at fixed intervals, by leveraging LDP.

## Related work

Recently, LDP has begun to attract attention as a promising way of guaranteeing individual privacy in the process of data collection. RAPPOR, which is one of the most representative data collection mechanisms based on LDA, was implemented in Google Chrome to collect user data [6]. *Fanti et al*. introduced a new algorithm to estimate the joint distribution between unknown variables by extending RAPPOR [7]. Apple has also leveraged LDP to collect user data, including new words, emojis, deeplinks, and lookup hints inside notes [8]. Recently, the differential privacy team in Apple introduced details of LDP deployment, which enabled the collection of large scale user data, including emojis, health data, and media playback preferences [9]. *Ding et al*. presented new LDP mechanisms for the repeated collection of counter data, which has been deloyed with Microsoft Windows Insiders [10]. LDP can be used for diverse application domains to collect user data while preserving privacy. [11–13] proposed a method for estimating heavy hitters over set-valued data. The proposed method in [12] consists of two phases: a candidate set selection phase that uses a portion of the privacy budget, and a refining phase that selects heavy hitters from the candidate set by leveraging the remaining privacy budget. In [13], LDP protocols to find out heavy hitters in a large domain was presented, where user are divided into groups, and each group reports a prefix of her value. [14] proposed the optimized local hashing protocol that can provides better accuracy than RAPPOR. Harmony, an advanced data analytics tool, based on LDP, supports the collection and analysis of user data in Samsung smartphones [15]. *Kim et al*. presented a method to estimate the density of a specific location in an indoor space by leveraging LDP [16]. [17] introduced a new technique for LDP to collect and track evolving local data, making it possible to maintain up-to-date statistics over time. In [18], the method for releasing low-order (2-way and 3-way) marginal statistic on population under LDP was developed.

With a growing need to share big data containing information regarding an individual entity, privacy-preserving data publishing (PPDP) has been extensively studied to share big data containing personal information for public use, while preserving the privacy of the individual. Various privacy models have been studied, including *k*-anonymity [19], *l*-diversity [20], and *t*-closeness [21]. Accordingly, research on privacy preserving data publishing methods for electronic health data has been actively conducted during the past decade. *Kim et al*. presented a delay-free method for publishing electronic health data streams, while preserving the privacy [22]. In [23], a utility-preserving anonymization method for PPDP was proposed. The proposed method in [23] preserves the utility of health data by inserting counterfeit record and creating catalog of the counterfeit records in the process of data anonymization. [24] presented the cost model that quantifies the trade-off between privacy and data utility in health data publishing. A comprehensive survey of privacy-preserving health data publishing can be found in [25].

## Background: Local differential privacy

Unlike differential privacy (DP) which was designed for the data-sharing purpose [26–33], LDP is the state-of-the-art approach to protect individual privacy in the process of data collection. The basic concept of LDP is that a data contributor adds carefully designed noises to her/his original data and sends the noisy data to a data collector, guaranteeing that the data contributor’s original data is not exposed to the outside of the data contributor devices. LDP is formally defined as follows: A randomized algorithm *A* satisfies *ϵ*-differential privacy, if and only if for (1) all pairs of data contributor’s data *v*_*i*_ and *v*_*j*_, and (2) any output *O* of *A*, the following equation holds [6]:

$$\begin{array}{c} \frac{Pr[A\left(v_{i}\right)=O]}{Pr[A\left(v_{j}\right)=O]}\leq e^{\epsilon } \end{array}$$

That is, regardless of the data that a data collector receives from a data contributor, the collector is not possible to speculate with high confidence whether the contributor has sent *v*_*i*_ or *v*_*j*_.

The privacy budget, *ϵ*, controls the level of privacy such that smaller values of *ϵ* enforce a stronger privacy guarantee, adding larger noises to the original data, while larger values of *ϵ* provide a weaker privacy guarantee, adding smaller noises to the original data. LDP follows the sequential composition property of differential privacy [12]. That is, given an available privacy budget *ϵ*, the data contributor can partition it into *w* smaller privacy budgets, *ϵ*_1_, *ϵ*_2_, ⋯, *ϵ*_*w*_, such that $\epsilon =\sum_{i=1}^{w}\epsilon_{i}$ and use each smaller privacy budget to report his/her local data to a data collector.

## Problem definition and straightforward solution

**Fig 2:**
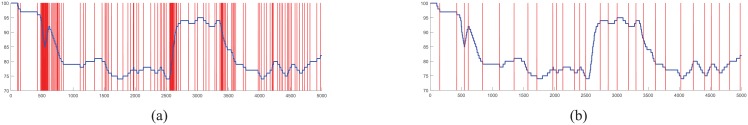
An example of salient points extracted from a given sequence. The blue curve represents the sequence of original health data. The point at which each red line parallel to the y-axis intersects the blue curve corresponds to a salient point.

Health data generated from wearable health devices are generally characterized as temporal data collected at fixed intervals. For example, the blue plot in Fig 2 represents the heart rate data of a person collected at fixed intervals over a certain period of time. Formally, let *U* = {*u*_1_, *u*_2_, ⋯, *u*_*w*_} be the set of users (i.e., data contributors). Here, *w* corresponds to the total number of users. Then, the health data stream of the *i*-th user, *u*_*i*_, can be represented as a sequence (or time series) *s*_*i*_ = ((*t*_1_, *x*_1_), (*t*_2_, *x*_2_), ⋯, (*t*_*n*_, *x*_*n*_)) of length *n*. Here, (*t*_*d*_, *x*_*d*_) represents the *d*-th point in the stream where *x*_*d*_ denotes the value measured by the wearable health device at timestamp, *t*_*d*_. We further assume that *x*_*d*_, which is measured by the specific sensor in a wearable health device, is within the predefined range [*x*_*min*_, *x*_*max*_].

In this paper, we focus on the scenario of collecting health data streams measured at the same fixed intervals during the same period (e.g., collecting heart rates measured every minute during business hours) using LDP. In this case, a straightforward solution is that each user, *u*_*i*_ ∈ *U*, partitions the privacy budget, *ϵ*, into *n* smaller privacy budgets, $\frac{\epsilon }{n}$, and uses each smaller privacy budget to generate a noisy sequence $s_{i}^{\prime }=(\left(t_{1},x_{1}^{\prime }\right),\left(t_{2},x_{2}^{\prime }\right),\cdots ,\left(t_{n},x_{n}^{\prime }\right))$. Here, $x_{d}^{\prime }$ is obtained using the Laplace mechanism as follows:

$$\begin{array}{c} x_{d}^{\prime }=x_{d}+Lap\left(\frac{\Delta s}{\epsilon /n}\right). \end{array}$$

Note that Δ*s* corresponds to the predefined sensitivity that is computed as Δ*s* = *x*_*max*_ − *x*_*min*_.

Let $S=\{s_{1}^{\prime },s_{2}^{\prime },\cdots s_{w}^{\prime }\}$ be a set of (noisy) sequences received from *w* users. Once the data collector received the noisy sequences from all the users, she/he can estimate the average value of *x*_*d*_ at timestamp, *t*_*d*_, by averaging all the noisy values of *x*_*d*_ in *S*:

$$\begin{array}{c} AVG_{est}\left(x_{d}\right)=\frac{1}{w}\times \sum_{s_{i}^{\prime }\in S}x_{d}^{\prime }. \end{array}$$

The expected error incurred by this estimation is known as $O(\frac{n}{\epsilon \sqrt{w}})$ [15], which is linearly proportional to the sequence length *n*. Thus, this scheme is not suitable when the sequence length, *n*, is large. Considering that the length of the sequence of a health data stream is typically large, this straightforward scheme is not suitable for our problem, owing to the high expected error.

## Proposed approach

**Fig 3:**
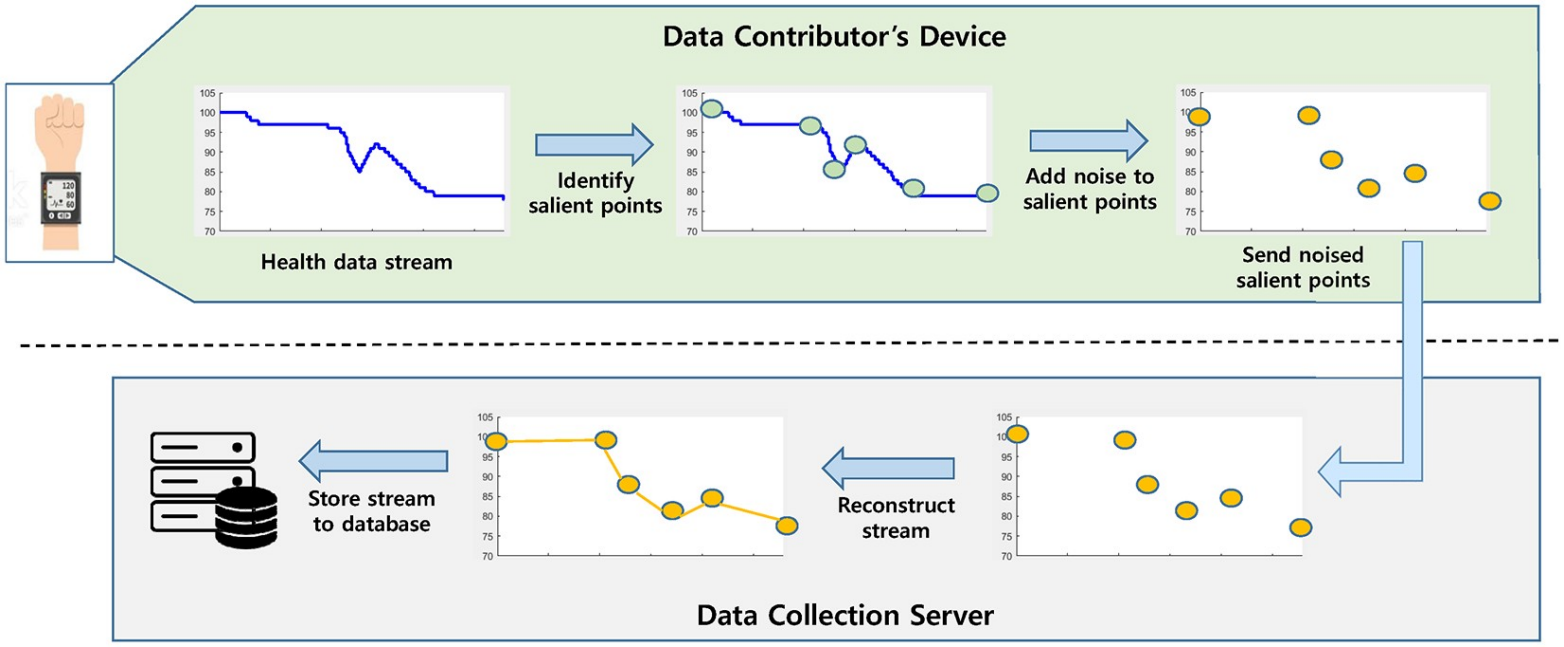
An overview of the proposed approach. The proposed approach can avoid a high expected error caused by the large sequence length by selecting and reporting a small amount of salient points to a data collector.

In this section, we describe the proposed scheme for collecting health data streams using LDP. As pointed earlier, the straightforward scheme may have an excessively high expected error, when the sequence length is large. To overcome this problem, in this paper, we propose a novel mechanism for collecting health data streams by leveraging LDP. Fig 3 shows an overview of the proposed approach that consists of the data contributor’s device-side and the data collection server-side processing.

- *Data contributor’s device:* The proposed method first identifies a small number of salient points from the sequence of a given health data stream, and then perturbs those points under LDP and reports noisy salient points to a data collection server.
- *Data collection server:* The proposed method reconstructs the sequence based on the noisy salient points received from the data contributor and stores it into a database for later use.

We note that the proposed approach avoids a high expected error caused by the large sequence length by selecting a small number of salient points from the health data stream and applying the LDP mechanism to those points alone. We now explain and describe each of these steps in detail.

### Data contributor’s device-side processing

#### Searching for salient points

Health data monitored by sensors in a wearable device are generally characterized as either remaining nearly constant or gradually increasing (or decreasing). For example, heart rate, oxygen saturation, and blood pressure of human beings remain nearly unchanged over long time periods of normal daily activities but gradually increase during unstable periods and then slowly decrease afterwards. Thus, given the sequence of health data, the objective of the first phase is to search for salient points where changes in the trends start.

Given the sequence of the health data stream of the *i*-th user, *s*_*i*_ = ((*t*_1_, *x*_1_), (*t*_2_, *x*_2_), ⋯, (*t*_*n*_, *x*_*n*_)), let *ds*_*i*_ = ((*t*_1_, *dx*_1_), (*t*_2_, *dx*_2_), ⋯, (*t*_*n*_, *dx*_*n*_)) be a corresponding sequence of the same length, obtained by taking a first-order derivative on *s*_*i*_. That is, *dx*_*h*_ (where 1 ≤ *h* ≤ *n*) is the first-order derivative of the sequence *s*_*i*_ at timestamp *t*_*h*_. By taking the first-order derivative of the sequence, we can differentiate points belonging to increasing or decreasing periods (i.e., *dx*_*h*_ < 0 or *dx*_*h*_ > 0) from the ones that are in constant periods (i.e., *dx*_*h*_ = 0).

As the objective of this phase is to search for salient points in the given sequence, we are interested in the case of *dx*_*h*_ ≠ 0 where 1 ≤ *h* ≤ *n*. However, given a sequence *s*_*i*_ of the length *n*, the number of points that satisfy the above condition can still be large. For example, Fig 2 illustrates the example of salient points extracted from a sequence of length 5,000. Here, the blue curve represents the sequence of original health data, *s*_*i*_. In the figure, the point at which each red line parallel to the y-axis intersects the blue curve corresponds to a salient point. Note that in Fig 2(a), salient points are simply obtained by searching for points that satisfy the condition, *dx*_*h*_ ≠ 0 (where 1 ≤ *h* ≤ *n*), after taking a first-order derivative on *s*_*i*_. As can be seen in this figure, the number of salient points identified using this scheme is still large.

**Fig 4:**
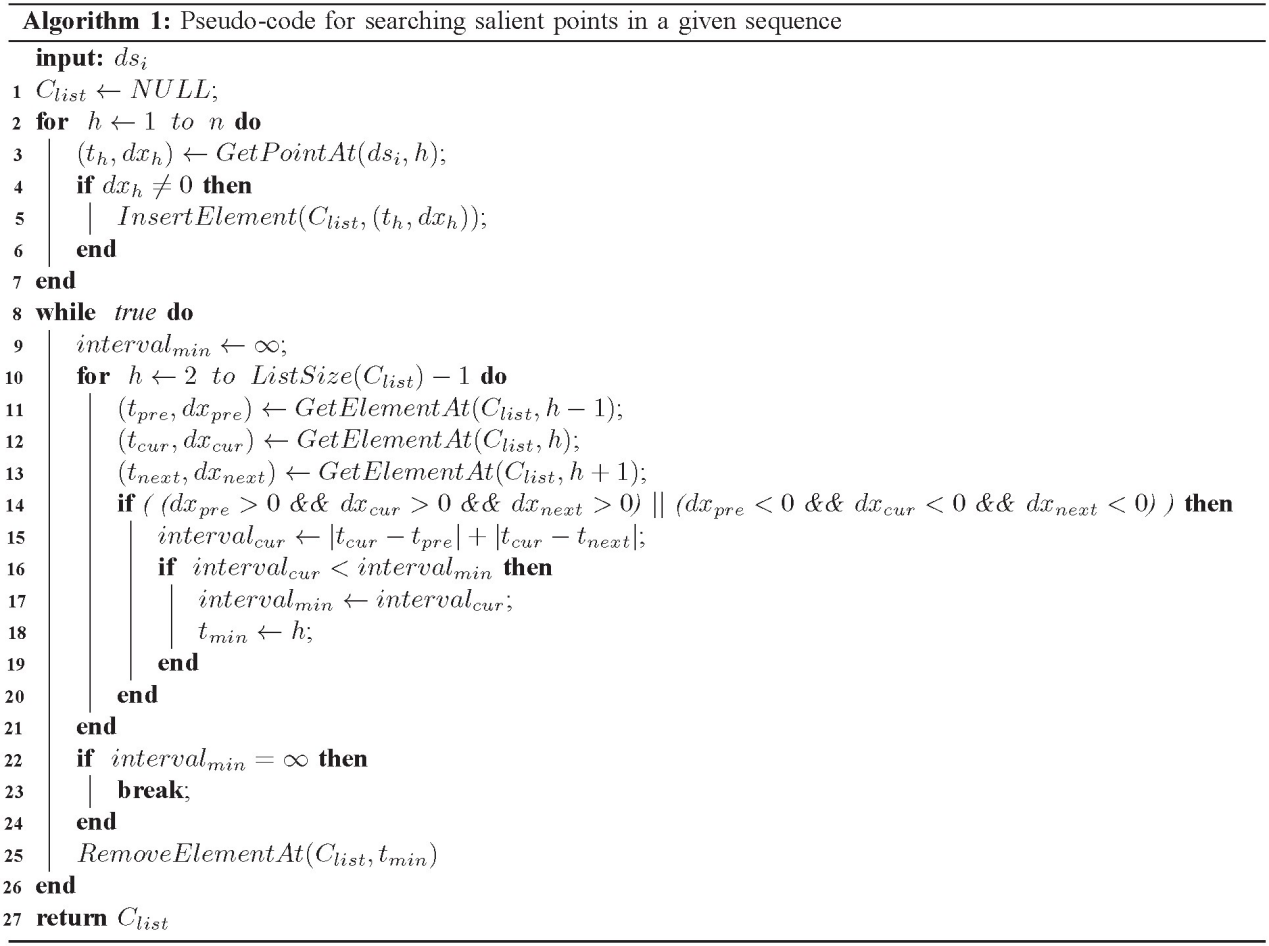
Pseudo-code for searching salient points in a given sequence.

Thus, the next step minimizes the number of salient points by iteratively merging time intervals belonging to the same trend (i.e, either an increasing or a decreasing trend), which is presented in Fig 4. The algorithm starts with the sequence *ds*_*i*_. In the initialization step, the algorithm sequentially scans each point in the sequence *ds*_*i*_ and inserts it into the list *C*_*list*_, if the first-order derivative at that point is not zero (lines 1-7). Note that the points in *C*_*list*_ is sorted by timestamp in ascending order because points in *ds*_*i*_ are scanned in timestamp order in the initialization step.

Given two adjacent points, (*t*_*h*_, *dx*_*h*_) and (*t*_*h*+1_, *dx*_*h*+1_), in *C*_*list*_, the corresponding time interval between these two point is defined as |*t*_*h*+1_ − *t*_*h*_|. The main idea of the algorithm in Fig 4 is to iteratively find and merge two adjacent time intervals belonging to the same trend (i.e, either an increasing or a decreasing trend), the summation of which is the shortest (lines 9-25). The iteration is terminated if the algorithm cannot find any two adjacent time intervals belonging to the same trend (lines 22-23). Finally, the algorithm returns the list, *C*_*list*_, that contains salient points. Fig 2(b) shows salient points obtained by further merging time intervals belonging to the same trend using the algorithm in Fig 4. As can be seen in the figure, the number of salient points can be significantly reduced by the method described in Fig 4.

#### Reporting noisy salient points

Once the salient points are identified, the next step is to add random noise to each salient point based on the LDP mechanism, and then send the noisy salient points to the data collection server. Let $SP_{i}=\left\{\left(t_{s_{1}},x_{s_{1}}\right),\left(t_{s_{2}},x_{s_{2}}\right),\cdots ,\left(t_{s_{r}},x_{s_{r}}\right)\right\}$ be the set of salient points extracted from the sequence *s*_*i*_ as explained in the previous phase. Let further assume that the timestamp of salient points in *SP* satisfies the condition, $t_{s_{1}}<t_{s_{2}}<\cdots <t_{s_{r}}$. Then, this phase first partitions the privacy budget, *ϵ*, into *r* smaller privacy budgets, such as *ϵ*_1_, *ϵ*_2_, ⋯, *ϵ*_*r*_, and then adds random noise, sampled from the Laplace distribution, to each salient point by consuming each partitioned privacy budget. As the probability density function of the Laplace distribution from which random noises are sampled is dependent on each privacy budget, *ϵ*_*h*_ (where 1 ≤ *h* ≤ *r*), in this paper, we introduce two different privacy budget partition schemes: uniform- and adaptive privacy budget partition.

- *Uniform privacy budget partition:* Given a privacy budget, *ϵ*, and a set of salient points $SP_{i}=\left\{\left(t_{s_{1}},x_{s_{1}}\right),\left(t_{s_{2}},x_{s_{2}}\right),\cdots ,\left(t_{s_{r}},x_{s_{r}}\right)\right\}$, this scheme uniformly partitions the privacy budget into *ϵ*_1_, *ϵ*_2_, ⋯, *ϵ*_*r*_ such that the following condition holds:
  $$\begin{array}{c} \epsilon_{1}=\epsilon_{2}=\cdots =\epsilon_{r}=\frac{\epsilon }{r}. \end{array}$$
- *Adaptive privacy budget partition:* Unlike uniform privacy budget partition, this scheme adaptively partitions a privacy budget based on the temporal scale of each salient point. As can be seen in Fig 2, each salient point covers a different temporal range. Let us consider three consecutive salient points, $\left(t_{s_{h-1}},x_{s_{h-1}}\right)$, $\left(t_{s_{h}},x_{s_{h}}\right)$, and $\left(t_{s_{h+1}},x_{s_{h+1}}\right)$. Then, the temporal scale, *μ*_*h*_, of the *h*-th salient point, $\left(t_{s_{h}},x_{s_{h}}\right)$, is computed as
  $$\begin{array}{c} \mu_{h}={\left(\frac{|t_{s_{h}}-t_{s_{h-1}}\left|+\right|t_{s_{h}}-t_{s_{h+1}}|}{2}\right)}^{\alpha }. \end{array}$$
  Here, *α* is a predefined system parameter. Let further assume that *μ*_*sum*_ is the summation of the temporal scale of each salient point in *SP*_*i*_ (i.e., *μ*_*sum*_ = ∑_1≤*h*≤*r*_ *μ*_*h*_). Then, this scheme partitions the privacy budget into *ϵ*_1_, *ϵ*_2_, ⋯, *ϵ*_*r*_ as following:
  $$\begin{array}{c} \epsilon_{h}=\epsilon \times \frac{\mu_{h}}{\mu_{sum}}. \end{array}$$
  Here, it is obvious that *ϵ* = ∑_1≤*h*≤*r*_ *ϵ*_*h*_. The intuition of this scheme is that larger the temporal scale of a salient point, more the privacy budget it is assigned to.

Once the privacy is partitioned into *r* smaller privacy budgets, next we use each smaller privacy budget to generate the set of noisy salient points, $SP_{i}^{\prime }=\left\{\left(t_{s_{1}},x_{s_{1}}^{\prime }\right),\left(t_{s_{2}},x_{s_{2}}^{\prime }\right),\cdots ,\left(t_{s_{r}},x_{s_{r}}^{\prime }\right)\right\}$. Here, $x_{s_{h}}^{\prime }$ is obtained using the Laplace mechanism as follows:

$$\begin{array}{c} x_{s_{h}}^{\prime }=x_{s_{h}}+Lap\left(\frac{\Delta s}{\epsilon_{h}}\right). \end{array}$$

That is, $x_{s_{h}}^{\prime }$ is computed by adding a random noise sampled from a Laplace distribution with mean *μ* = 0 and scale $b=\frac{\Delta s}{\epsilon_{h}}$ to the original value of $x_{s_{h}}$. Note that as explained earlier, Δ*s* corresponds to the predefined sensitivity that is computed as Δ*s* = *x*_*max*_ − *x*_*min*_.

Note that in the case of uniform privacy budget partition, the same probability density function of the Laplace distribution is used for adding a random noise to each salient point, owing to the condition, *ϵ*_1_ = *ϵ*_2_ = ⋯ = *ϵ*_*r*_. On the other hands, in the case of adaptive privacy budget partition, different probability density functions of the Laplace distribution are used for each salient point because the privacy budgets allocated for perturbing each salient point are different from each other. As the Laplace scale factor $b=\frac{\Delta s}{\epsilon_{h}}$ decreases (increases), which corresponds to the case where the privacy budget *ϵ*_*h*_ increases (decreases), the magnitude of the noise drawn from the Laplace distribution tends to decrease (increase). Thus, the adaptive privacy budget partition scheme ensures that smaller noises are added to more important salient points having larger temporal scales. However, less important salient points whose temporal scale is small are perturbed with larger noises.

Finally, the set of noisy salient points, $SP_{i}^{\prime }=\left\{\left(t_{s_{1}},x_{s_{1}}^{\prime }\right),\left(t_{s_{2}},x_{s_{2}}^{\prime }\right),\cdots ,\left(t_{s_{r}},x_{s_{r}}^{\prime }\right)\right\}$, is directly sent to a data collection server, guaranteeing that the original data of the data contributor is not exposed to outside users. In this paper, we assume that the set of noisy salient points is transmitted through secure channels established between the data contributor’s device and the data collection server. We also note that sending noisy salient points to a data collection server may raise a possible privacy issue in certain applications. That is, by observing the reported timestamps, the adversary may infer certain information about the pattern of the data contributor’s health data stream. One possible solution to such privacy concerns is to add dummy salient points to the set of noisy salient points, and thus, the adversary cannot differentiate between real and dummy salient points.

### Data collection server-side processing

Upon receiving the set of noisy salient points, $SP_{i}^{\prime }=\left\{\left(t_{s_{1}},x_{s_{1}}^{\prime }\right),\left(t_{s_{2}},x_{s_{2}}^{\prime }\right),\cdots ,\left(t_{s_{r}},x_{s_{r}}^{\prime }\right)\right\}$ from the *i*-th user, *u*_*i*_, the first step of a data collection server-side processing is to reconstruct the health data stream based on the received salient points. In this subsection, we present two different methods to rebuild the health data stream: linear and nonlinear estimation.

**Fig 5:**
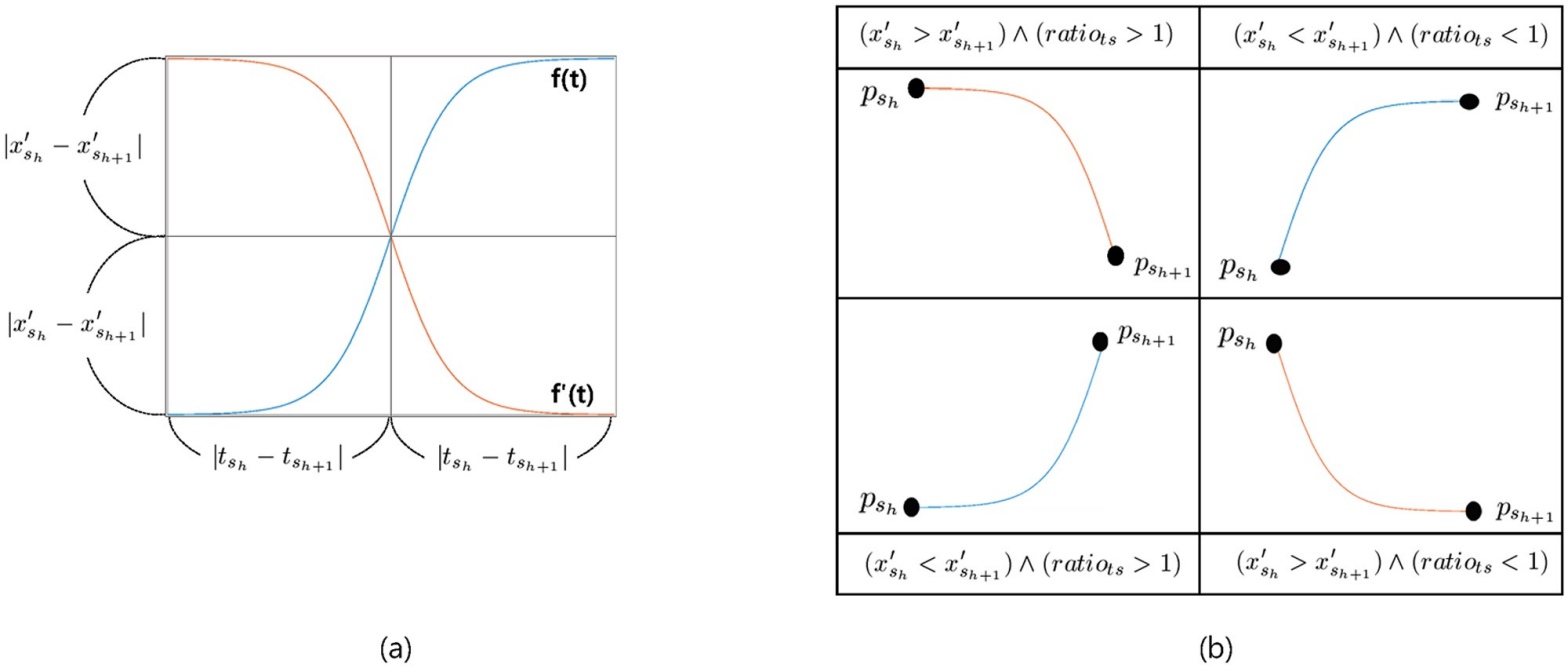
(a) Logistic curve and its symmetric curve for two salient points, $p_{s_{h}}=\left(t_{s_{h}},x_{s_{h}}^{\prime }\right)$ and $p_{s_{h+1}}=\left(t_{s_{h+1}},x_{s_{h+1}}^{\prime }\right)$, and (b) four different curves that are used to rebuild a stream segment depending on the values of *μ*_*ratio*_ and $\left(x_{s_{h}}^{\prime }-x_{s_{h+1}}^{\prime }\right)$.

- *Linear estimation:* The first scheme is to use a straight line connecting two adjacent salient points to rebuild the data stream. Let us consider the case of two adjacent salient points, $\left(t_{s_{h}},x_{s_{h}}^{\prime }\right)\in SP_{i}^{\prime }$, and $\left(t_{s_{h+1}},x_{s_{h+1}}^{\prime }\right)\in SP_{i}^{\prime }$. Then, the slope, *a*, and the y-intercept, *b*, of the straight line connecting these saline points are respectively computed as
  $$\begin{array}{c} a=\frac{x_{s_{h+1}}^{\prime }-x_{s_{h}}^{\prime }}{t_{s_{h+1}}-t_{s_{h}}},\;\;\;b=x_{s_{h}}^{\prime }-a\times t_{s_{h}}. \end{array}$$
  Then, a stream segment between $t_{s_{h}}$ and $t_{s_{h+1}}$ is estimated with the line connecting these two adjacent salient points.
- *Nonlinear estimation:* Unlike the first method, the second approach exploits prior information regarding the privacy budget partition of the data contributor’s device-side processing. Given two adjacent salient points, $p_{s_{h}}=\left(t_{s_{h}},x_{s_{h}}^{\prime }\right)$ and $p_{s_{h+1}}=\left(t_{s_{h+1}},x_{s_{h+1}}^{\prime }\right)$, the ratio of time scale of these two points is computed as $\mu_{ratio}=\frac{\mu_{s_{h}}}{\mu_{s_{h+1}}}$. In the case of adaptive privacy budget partition scheme, if *μ*_*ratio*_ is greater than 1, it is likely that the gap between $x_{s_{h}}^{\prime }$ and the corresponding original value (i.e, $x_{s_{h}}$) is smaller than the gap between $x_{s_{h+1}}^{\prime }$ and the corresponding original value (i.e, $x_{s_{h+1}}$). This is because more privacy budget is used for adding random noise to $x_{s_{h}}$ than to $x_{s_{h+1}}$. In such scenarios, a more reasonable solution to rebuild a stream segment between $t_{s_{h}}$ and $t_{s_{h+1}}$ is to leverage a nonlinear curve biased to $x_{s_{h}}$. The case where *μ*_*ratio*_ < 1 is similarly explained. If *μ*_*ratio*_ = 1, then the linear estimation scheme is used to rebuild a stream segment.
  Based on the above intuition, we use the following logistic function, *f*(*t*), and its symmetric function, *f*′(*t*) (Fig 5(a)):
  $$\begin{array}{c} f\left(t\right)=\frac{L}{1+e^{-\beta t}},\;\;f^{\prime }\left(t\right)=L-\frac{L}{1+e^{-\beta t}}, \end{array}$$
  where the curve’s maximum value, *L*, is defined as $2\times |x_{s_{h}}^{\prime }-x_{s_{h+1}}^{\prime }|$ and the steepness of the curve, *β*, is a predefined system parameter. Then, as can be seen in Fig 5(a), given two functions, *f*(*t*) and *f*′(*t*), the entire space is divided into four subspaces, generating four different biased curves that are used to rebuild the stream segment between $t_{s_{h}}$ and $t_{s_{h+1}}$, depending on the values of *μ*_*ratio*_ and $\left(x_{s_{h}}^{\prime }-x_{s_{h+1}}^{\prime }\right)$. For example, if *μ*_*ratio*_ is greater than 1, the nonlinear curve biased to $x_{s_{h}}^{\prime }$ is used to rebuild the stream segment between $t_{s_{h}}$ and $t_{s_{h+1}}$, which corresponds to the top-left and bottom-left cases in Fig 5(b). On the other hands, if *μ*_*ratio*_ is less than 1, the nonlinear curve biased to $x_{s_{h+1}}^{\prime }$ is used to rebuild the stream segment between $t_{s_{h}}$ and $t_{s_{h+1}}$ which corresponds to the top-right and bottom-right cases in the figure.

Then, the *i*-th data contributor’s reconstructed health data stream, $s_{i}^{\prime }=(\left(t_{1},x_{1}^{\prime }\right),\left(t_{2},x_{2}^{\prime }\right),\cdots ,\left(t_{n},x_{n}^{\prime }\right))$, is stored into a database. Let $S=\{s_{1}^{\prime },s_{2}^{\prime },\cdots s_{w}^{\prime }\}$ be the set of sequences stored in the database. Then, the average value of *x*_*d*_ at the timestamp, *t*_*d*_, is estimated as

$$\begin{array}{c} AVG_{est}\left(x_{d}\right)=\frac{1}{w}\times \sum_{s_{i}^{\prime }\in S}x_{d}^{\prime }. \end{array}$$

We note that unlike the straightforward solution, the proposed approach avoids high expected errors caused by large sequence lengths, as such the data contributor reports a small number of salient points to a data collector who then estimates the original health data stream based on the salient points received from the data contributor.

## Experiment

In this section, we describe the experiments we carried out to evaluate the effectiveness of the proposed approach. First we describe the experimental setup and thereafter we discuss the results.

### Experimental setup

We evaluated the proposed approach with the PAMAP2 physical activity monitoring dataset [34] that contains the set of sensory data from nine subjects wearing three inertial measurement units and a heart rate monitor. We note that the PAMAP2 dataset contains a heart rate monitoring dataset that is collected using sensors, which is well suited for our experiments. We first extracted eight heart rate data streams whose length is 3,000 from the PAMAP2 datasets. To investigate the effect of the collected data size on the performance, we generated large synthetic data sets using these eight real heart rate data streams. Given a real heart rate data stream, a synthetic data stream was generated by adding a random noise, which was sampled from a Laplace distribution with mean *μ* = 0 and scale *b* = 1, to each point in the real heart rate data stream. For experiments in this section, we generated four different sizes of data sets: 80K, 160K, 320K, and 640K.

In the experiments, we report results for the following alternatives:

- *ldp*_*full* corresponds to the straightforward solution that reports all points in a health data stream.
- *ldp*_*ul* is the proposed approach of using the uniform privacy budget partition scheme (data contributor’s device-side) and linear estimation method (data collection server-side).
- *ldp*_*al* is the proposed approach of using the adaptive privacy budget partition scheme and linear estimation method.
- *ldp*_*an* is the proposed approach of using the adaptive privacy budget partition scheme and nonlinear estimation method.
- *ldp*_*rl* corresponds to a method based on randomly selected salient points and the linear estimation method. That is, unlike *ldp*_*ul*, *ldp*_*al*, and *ldp*_*an*, in which salient points are identified by the proposed algorithm shown in Fig 4, given the number of salient points (*sp*_*num*_), *ldp*_*rl* randomly (but uniformly) selects *sp*_*num*_ points from a health data stream and uses these randomly selected points as salient points. Here, *sp*_*num*_, is determined by averaging the number of salient points identified from each health data stream used in the experiment using the proposed algorithm shown in Fig 4. Note that the purpose of reporting the results of *ldp*_*rl* is to experimentally evaluate the usefulness of the proposed salient point searching algorithm.

To compare the five schemes, we use an error rate, *e*:

$$\begin{array}{c} e=\frac{1}{n}\times \sum_{d=1}^{n}\frac{|AVG_{actual}\left(x_{d}\right)-AVG_{est}\left(x_{d}\right)|}{AVG_{actual}\left(x_{d}\right)}. \end{array}$$

Here, *AVG*_*est*_(*x*_*d*_) and *AVG*_*actual*_(*x*_*d*_) is the estimated- and the actual average value of *x*_*d*_ at the timestamp, *t*_*d*_, respectively, and *n* denotes the sequence length. The parameters, *α*, and *β*, are set to 0.5, which provides considerably good estimation performances. We run each experiment three times and the error rates reported in the experiment are the averages of all runs.

### Results and discussion

**Fig 6:**
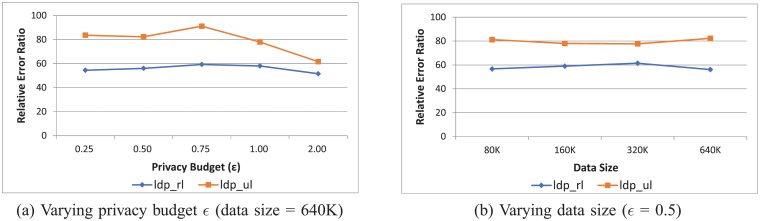
Relative error ratio for varying privacy budget *ϵ* and data size.

We first compare the error rate of two different categories of methods: *ldp*_*full* that uses a privacy budget to report all points in a health data stream under LDP and *ldp*_*ul* and *ldp*_*rl* that consume a privacy budget to report only salient points (or randomly selected points) under LDP. To compare these three schemes, in Fig 6, we use the relative error ratio:

$$\begin{array}{c} \frac{error\;rate\;of\;ldp\_full}{error\;rate\;of\;ldp\_ul\;(or\;ldp\_rl)}. \end{array}$$

The relative error ratio being greater than 1 means that the approach reporting only salient points (or randomly selected points) outperforms the method reporting all points. In Fig 6(a), the privacy budget, *ϵ*, varies from 0.25 to 2.0, while the data size is set to 640K. On the other hands, in Fig 6(b), the data size varies from 80K to 640K, while the privacy budget is fixed at 0.5. As can be seen from the figure, both *ldp*_*ul* and *ldp*_*rl* significantly outperform *ldp*_*full*. In particular, with the proposed *ldp*_*ul*, performance gains from 60X to 90X are possible. The experiment results in Fig 6 verify that when collecting health data streams, characterized as long in length, under LDP, it is much more effective for reporting only small number of points than reporting all points in the stream. The experimental results in Fig 6 further show that the proposed *ldp*_*ul* outperforms *ldp*_*rl*, at all privacy budgets and data sizes. This verifies that given a health data stream, it is more effective to report carefully selected salient points using the method presented in this paper than randomly selected points.

**Fig 7:**
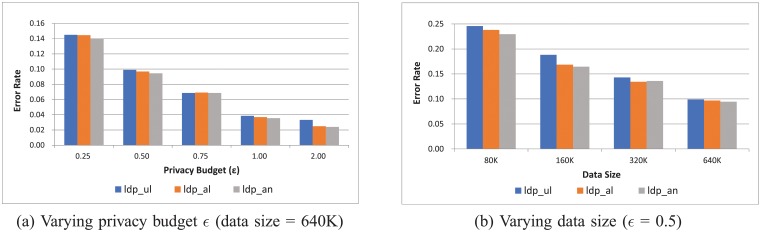
Error rate for varying privacy budget *ϵ* and data size.

Fig 7 shows the error rate for varying (a) privacy budget, *ϵ*, and (b) data size for three different schemes, *ldp*_*ul*, *ldp*_*al*, and *ldp*_*an*, proposed in the paper. The data size is set to 640K in Fig 7(a) and the privacy budget is fixed to 0.5 in Fig 7(b). Key observations based on Fig 7 can be summarized as follows:

- As expected, the error rate decreases, as the data size increases, which indicates that the proposed approach well exploits the collected data.
- As the privacy budget, *ϵ*, increases, the error rate decreases. This is because, as the privacy budget increases, noises added by the data contributor’s device-side decrease, and thus the level of privacy decreases. This, in turn, results in increased estimation accuracy at the data collection server-side.
- Among three different schemes, *ldp*_*an*, which is based on the adaptive privacy budget partition scheme and nonlinear estimation method, produces slightly better results than the other approaches, *ldp*_*ul* and *ldp*_*al*, which implies that *ldp*_*an* is suitable for applications that require high level of estimation accuracy.

**Fig 8:**
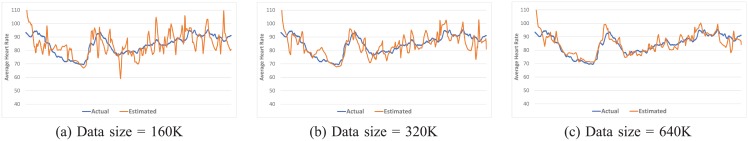
Actual vs. estimated stream of the average heart rates for varying data size (*ϵ* = 1.0).

To further investigate the effects of collected data size on the estimation accuracy, we plot the stream of average heart rates for varying data sizes in Fig 8. In this experiment, data size varies from 160K to 640K, while the value of *ϵ* is fixed to 1.0. In this experiment, the estimated stream is obtained using *ldp*_*an*. As the collected data size increases, the estimated stream (orange plot in Fig 8) obtained with *ldp*_*an* becomes similar to the actual one (blue plot in Fig 8). With a 160K collected data set, a good estimation cannot be achieved because the collected data size is insufficient. However, with a 640K collected data set, the proposed approach in this paper produces a fairly good estimation. This experiment result indicates that the proposed method well exploits the collected data set.

## Conclusion and future work

In this study, we developed a novel mechanism to collect individual health data streams generated from various smart healthcare sensors in a privacy-preserving manner using LDP. Our proposed approach first identifies a small number of salient data points from an entire health data stream of a data contributor, perturbs these identified salient data points under LDP, and then reports the perturbed salient data to a data collector, instead of reporting all the data in the stream. Furthermore, we presented an effective method that enables a data collector to reconstruct the health data stream from the perturbed data set received from the data contributor. Experiments demonstrated that the proposed method provides a significant improvement in results when compared with the straightforward solutions to this problem. In a future work, we are planning to extend the proposed data collection framework such that it is possible to compute marginal statistics with multiple types of health data streams.
